# MEFV M694V mutation has a role in susceptibility to ankylosing spondylitis: A meta-analysis

**DOI:** 10.1371/journal.pone.0182967

**Published:** 2017-08-11

**Authors:** Linqing Zhong, Hongmei Song, Wei Wang, Ji Li, Mingsheng Ma

**Affiliations:** Department of Pediatrics, Peking Union Medical College Hospital, Chinese Academy of Medical Sciences and Peking Union Medical College, Beijing, China; Oregon Health and Science University, UNITED STATES

## Abstract

**Objective:**

The aim of the current study was to determine the contributions of several common mutations in the Mediterranean fever (MEFV) gene, namely, E148Q, M680I, M694V and V726A, to ankylosing spondylitis (AS) susceptibility.

**Methods:**

Two investigators independently searched the literature regarding the association of MEFV with AS in the PubMed, EMBASE, Web of Science, and Scopus databases. They independently selected eligible articles and then extracted data from the included studies. The associations between MEFV mutations and AS risk were assessed with odds ratios (ORs) and 95% confidence intervals (95% CI). Further analyses were conducted with STATA 12.0 software (Stata Corp.; College Station, Texas, USA).

**Results:**

Four mutations (E148Q, M680I, M694V and V726A) were genotyped in 869 AS cases and 879 controls from the 8 eligible studies. Of the four mutations, M694V (pooled OR: 3.330, 95% CI: 2.129–5.208) was found to be associated with AS through overall analysis. However, the other mutations demonstrated no relation with AS (pooled ORs: 1.295, 1.258, 1.778; 95% CI: 0.886–1.891, 0.688–2.298 and 0.938–3.371). No significant publication bias was discovered in the meta-analysis.

**Conclusions:**

The present study indicates that the MEFV M694V mutation may contribute to the pathogenesis of AS. The associations between the other mutations and AS need to be validated with more relevant and well-designed studies.

## Introduction

Ankylosing spondylitis (AS) is a chronic inflammatory disease involving the axial skeleton, and its main clinical manifestations are back pain and progressive stiffness of the spine. AS typically develops in young adults with a peak age of onset between 20 and 30 years. Many AS patients have poor outcomes, and they may gradually progress to spinal fusion with hyperkyphosis. Patients develop work disability [1] and an impaired quality of life (QoL) [2] and are also at a high risk of adverse events such as spinal fracture [3]. The mean prevalence of AS is relatively high all over the world, ranging from 7.4 to 31.9 per 10,000 [4]. Estimates of the prevalence of ankylosing spondylitis in the United States even reach 0.5% [5]. Moreover, AS has significant economic implications for individuals and society [6–8]. Therefore, it is urgent to study the pathogenesis and treatment of ankylosing spondylitis. However, the pathogenesis of ankylosing spondylitis is inconclusive. Genetic influences are considered particularly important in the pathogenesis of AS. The human leukocyte antigen (HLA)-B27 has been regarded as a dominant susceptibility gene for ankylosing spondylitis [9, 10], but HLA-B27 positivity is relatively low in some conditions [11–13]. Furthermore, only a few of the patients who are positive for HLA-B27 develop AS [14, 15]. With the deepening of cognition, there have been an increasing number of studies concerning the influence of non-HLA genes, including IL-23R, IL-1, IL12-B, TNF-α, ERAP1, etc. [16–22].

The Mediterranean fever (MEFV) gene is located on the short arm of chromosome 16 and is known to be responsible for familial Mediterranean fever (FMF). FMF is a hereditary autoinflammatory disorder that is characterized by recurrent episodes of fever and serositis. Enthesopathy and sacroiliitis occur in some FMF patients [23–25], and up to 7.5% of FMF patients are simultaneously diagnosed with AS [26]. Furthermore, linkage has been observed between the AS bath ankylosing spondylitis disease activity index (BASDAI) and chromosome 16p in a whole-genome linkage scan [27]. Thus, several studies have investigated the association between AS and the most common mutations of MEFV, but the conclusions are uncertain and controversial. The aim of the present study was to comprehensively observe the contribution of several common mutations in the MEFV gene, namely, E148Q, M680I, M694V and V726A, to ankylosing spondylitis susceptibility.

## Materials and methods

### Data sources

**Fig 1:**
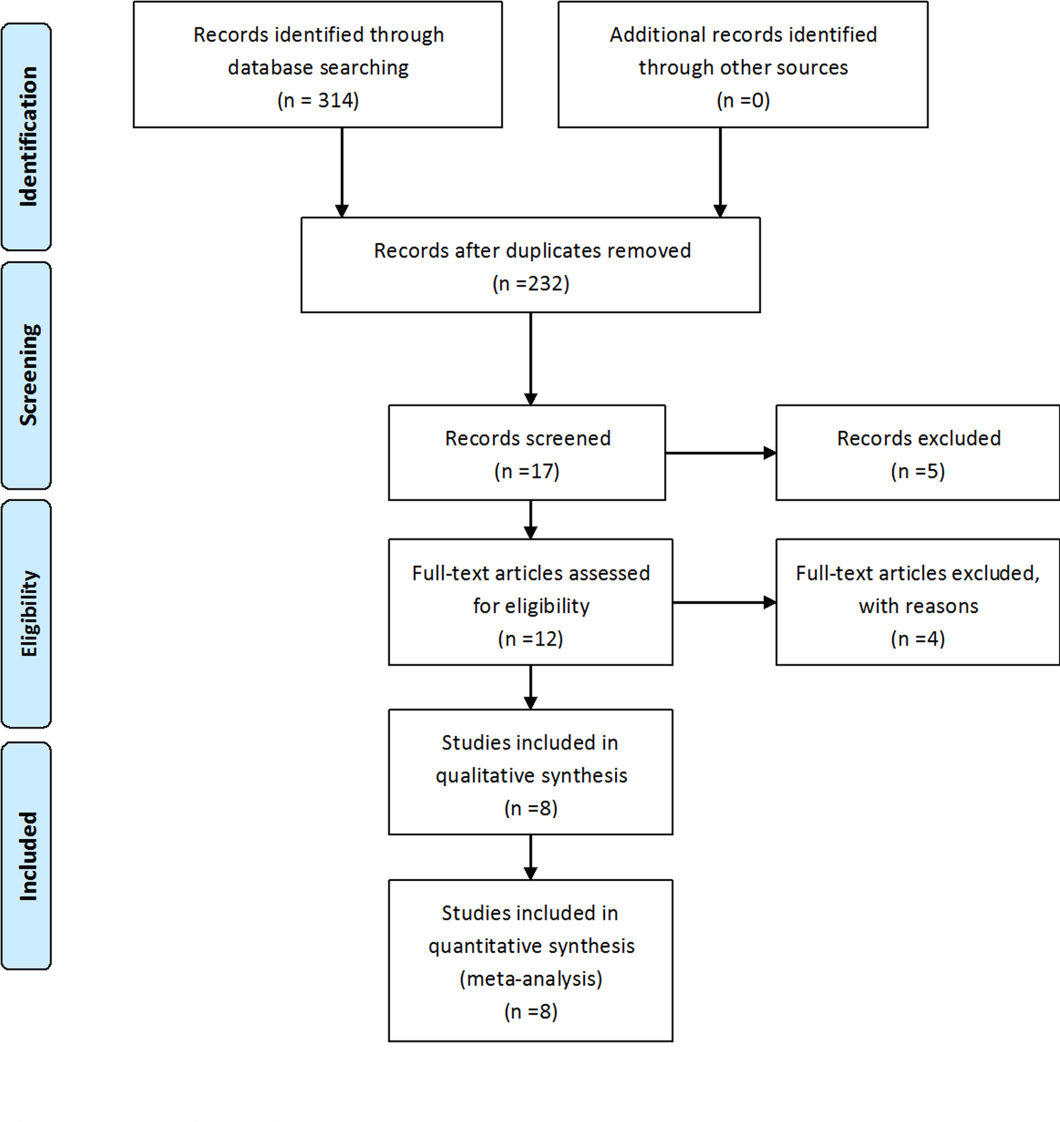
Flowchart of the selection of the 8 eligible studies in the meta-analysis.

A comprehensive literature search was performed in the PubMed, EMBASE, Web of Science, and Scopus databases up to the date of December 31, 2016. Full text searches were used in the PubMed, EMBASE and Scopus databases, while a medical subject headings (MeSH) search was applied in the Web of Science database. The keywords were “ankylosing spondylitis” and “MEFV”. No restrictions were imposed on the type of studies. The study selection process is illustrated in Fig 1.

### Inclusion and exclusion criteria

The included studies met the following criteria: 1) articles published before Dec 31, 2016; 2) case-control studies concerning the correlation between MEFV mutations and AS; and 3) studies with adequate data that allowed for the calculation of ORs with 95% CIs. The exclusion criteria were as follows: 1) case reports or case series; 2) studies without controls; 3) studies without available data; and 4) duplicate studies.

### Data extraction

The following items were extracted independently by two researchers (Linqing Zhong and Wei Wang): 1) characteristics of included studies, i.e., the title, the first author, publication year, sample size, study population, and genotyping methods; and 2) the frequencies of each MEFV mutation (i.e., E148Q, M680I, M694V and V726A) in the AS patients and controls. Discrepancies between these two investigators were resolved by discussion or consultation with a third researcher (Hongmei Song).

### Quality assessment

The two researchers (Linqing Zhong and Wei Wang) independently evaluated the qualities of the included studies with the Newcastle Ottawa Scale (NOS) [28]. The NOS form includes the following 3 sections: the selection of cases and controls, the comparability between the groups, and the ascertainment of exposure. A star rating system was applied, and each could be awarded a maximum of nine stars altogether.

### Statistical methods

The meta-analysis was performed with STATA 12.0 software (Stata Corp.; College Station, Texas, USA). The relevance of the MEFV mutations with AS was assessed with the pooled ORs and 95% CIs. The heterogeneity between studies was estimated with the Q-test (statistically significant heterogeneity existed when the p-value < 0.10) and the I^2^ statistic (25%, 50% and 75% were regarded as the cut-off points for low, moderate and high heterogeneity, respectively). A fixed-effects model (the Mantel-Haenszel method) was employed when I^2^<25%, otherwise a random-effects model (the Mantel-Haenszel method) was used. Begg’s funnel plots and Egger’s test were applied to assess the risk of publication bias, and the p-value was set at 0.10. Sensitivity analysis was implemented to determine the influence of each study on the pooled OR. The Hardy–Weinberg equilibriums (HWIs) of the controls were assessed with the STATA software. P-values < 0.05 were considered statistically significant. Subgroup analyses concerning the different populations were not involved in this study.

## Results

### Characteristics of the studies

A total of 314 articles were acquired from 4 databases (i.e., PubMed: 21, EMBASE: 49, Web of Science: 38, and Scopus: 206). A total of 297 articles were initially excluded after review of the titles and abstracts; of these, 82 were duplicate articles and 215 were not related to the relevant topic. The remaining 17 articles were reviewed in full text, and then 5 meeting reports were excluded. Among these 5, 4 were conducted by the same researchers as the rest of the studies, and the full text of one article was not available. Ultimately, one case report and 3 studies without control groups were ruled out. Finally, eight eligible case-control studies with 869 cases and 879 controls were included in the present meta-analysis. The characteristics of each study are presented in Table 1.

**Table 1:** Characteristics of the included studies.

Author	Year	Population	No. of case	No. of control	Diagnostic criteria	Genotyped method	Scores of NOS
Cosan F [29]	2010	Turkish	193	103	MNY	PCR-RFLP	8
Durmus D [30]	2009	Turkish	80	85	MNY	FMF Strip Assay	7
Yigit S [31]	2012	Turkish	103	120	MNY	PCR-RFLP	7
Kaya S [32]	2015	Turkish	34	35	MNY	FMF Strip Assay	6
Akkoc N [33]	2010	Turkish	62	50	MNY	REED & direct sequencing	4
He C^a^^,^^b^ [34]	2014	Chinese	200	200	MNY	Direct sequencing	8
Yilmaz E [16]	2014	Turkish	100	100	NA	FMF strip assay	6
Aslı Tufan [35]	2014	Turkish	97	186	MNY	ARMS-PCR & PCR-RFLP	7

MNY: the modified New York criteria for ankylosing spondylitis; PCR-RFLP: polymerase chain reaction-restriction fragment length polymorphisms; FMF: familial Mediterranean fever; REED: restriction endonuclease enzyme digestion; ARMS-PCR: amplification refractory mutation system-polymerase chain reaction.

^a^The E148Q mutation was not included in He C’s study.

^b^The V726A mutation was not found in either the AS or control group.

### MEFV mutations and AS

#### E148Q

The association of E148Q with the risk of AS was investigated in 7 independent studies. The frequency of E148Q was 65 of 1338 alleles in the AS patients (4.86%) and 54 of 1358 alleles in the controls (3.98%). No significant heterogeneity was identified by the Q-test and I^2^ statistics; therefore, a fixed-effects model was adopted for the analysis of the association of E148Q with AS susceptibility (pooled OR: 1.295, 95% CI: 0.886–1.891).

#### M680I

All of the included studies explored the relationship between M680I and AS. The M680I incidence rates were 23/1738 (1.32%) and 19/1758 (1.08%) in the AS patients and controls, respectively. A fixed-effects model was applied to estimate the relevance of the association of M680I with AS, and no significant difference was found between the groups (pooled OR: 1.258, 95% CI: 0.688–2.298).

#### M694V

**Fig 2:**
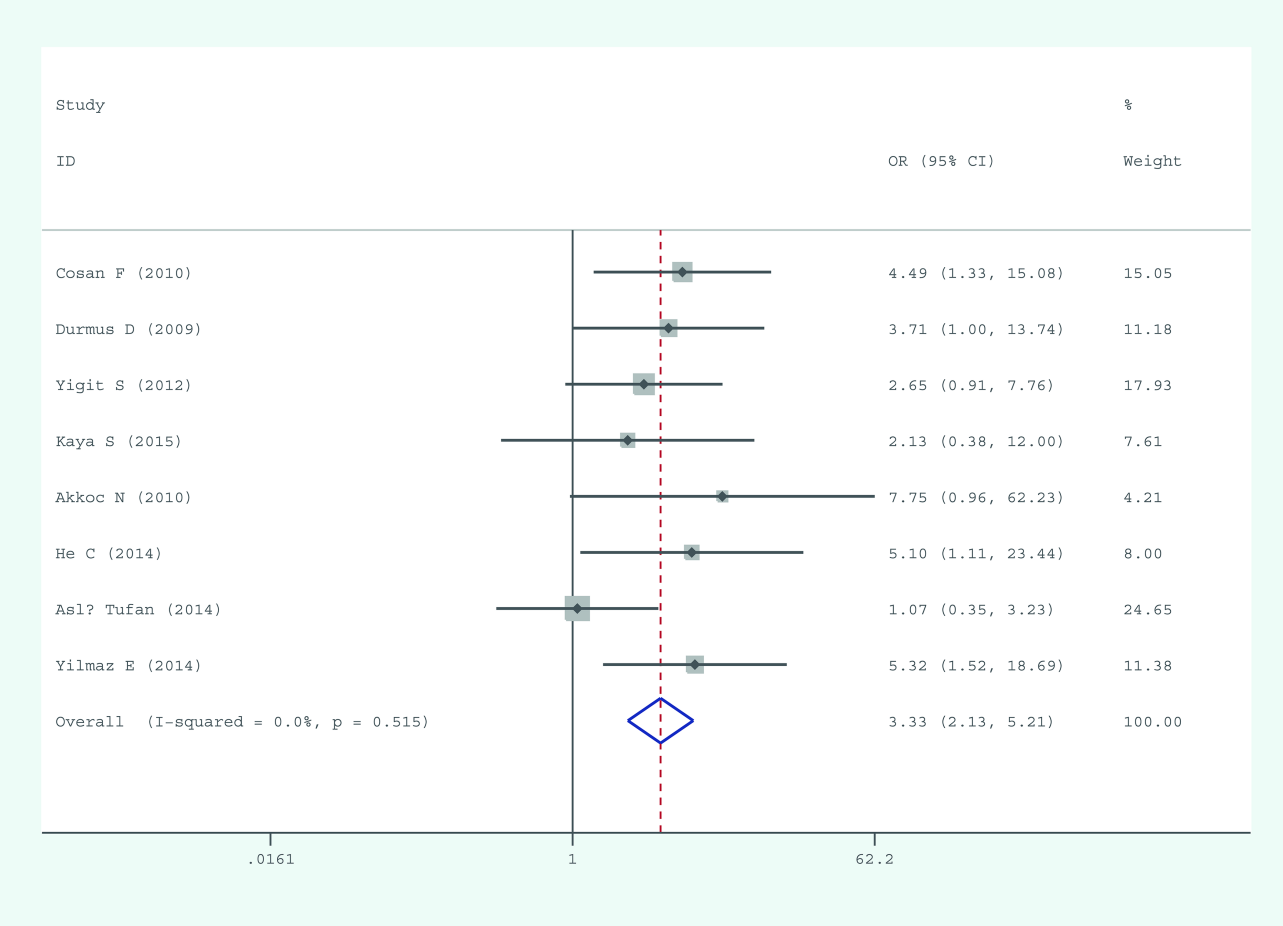
Forest plot of the M694V mutation.

All eligible studies assessed risk of AS in association with M694V. The M694V mutation occurred at a distinctly higher frequency in the AS patients (88/1738; 5.06%) than in the controls (28/1758; 1.59%). A fixed-effects model was used to estimate the overall OR and 95% CI because no significant heterogeneity was found. The result suggested that M694V might be a risk factor for AS (pooled OR: 3.330, 95% CI: 2.129–5.208). A forest plot of the association between the M694V mutation and AS is presented in Fig 2.

#### V726A

The connection between V726A with AS was also evaluated in all eight studies. However, one study was excluded from the meta-analysis because it had the same incidence rate for both groups. No significant statistical correlation was discovered (pooled OR: 1.778, 95% CI: 0.938–3.371) with a fixed-effects model. The frequency of each mutation in the cases and controls are shown in Table 2. Separate forest plots of the associations of E148Q, M680I and V726A with AS can be found in S1–S3 Figs.

**Table 2:** Frequencies of each mutation in case and control groups.

Author	Mutation	No. of case	No. of control	case			control			HWI
				(+)	(-)	total	(+)	(-)	total	
Cosan F	E148Q	193	103	17	369	386	7	199	206	Y
Durmus D	E148Q	80	85	7	153	160	4	166	170	Y
Yigit S	E148Q	103	120	9	197	206	5	235	240	Y
Kaya S	E148Q	34	35	7	61	68	2	68	70	Y
Akkoc N	E148Q	62	50	3	121	124	4	96	100	Y
Yilmaz E	E148Q	100	100	12	188	200	12	188	200	Y
Aslı Tufan	E148Q	97	186	10	184	194	20	352	372	Y
Cosan F	M680I	193	103	3	383	386	1	205	206	Y
Durmus D	M680I	80	85	4	156	160	4	166	170	Y
Yigit S	M680I	103	120	8	198	206	5	235	240	Y
Kaya S	M680I	34	35	1	67	68	0	70	70	Y
Akkoc N	M680I	62	50	3	121	124	1	99	100	Y
He C	M680I	200	200	2	398	400	1	399	400	Y
Yilmaz E	M680I	100	100	0	200	200	5	195	200	Y
Aslı Tufan	M680I	97	186	2	192	194	2	370	372	Y
Cosan F	M694V	193	103	24	362	386	3	203	206	Y
Durmus D	M694V	80	85	10	150	160	3	167	170	Y
Yigit S	M694V	103	120	11	195	206	5	235	240	Y
Kaya S	M694V	34	35	4	64	68	2	68	70	Y
Akkoc N	M694V	62	50	9	115	124	1	99	100	Y
He C	M694V	200	200	10	390	400	2	398	400	Y
Yilmaz E	M694V	100	100	15	185	200	3	197	200	Y
Aslı Tufan	M694V	97	186	5	189	194	9	363	372	Y
Cosan F	V726A	193	103	7	379	386	1	205	206	Y
Durmus D	V726A	80	85	2	158	160	2	168	170	Y
Yigit S	V726A	103	120	5	201	206	3	237	240	Y
Kaya S	V726A	34	35	2	66	68	0	70	70	Y
Akkoc N	V726A	62	50	3	121	124	2	98	100	Y
He C	V726A	200	200	0	400	400	0	400	400	Y
Yilmaz E	V726A	100	100	6	194	200	2	198	200	Y
Aslı Tufan	V726A	97	186	2	192	194	6	366	372	Y

### Publication bias and sensitivity analysis

**Fig 3:**
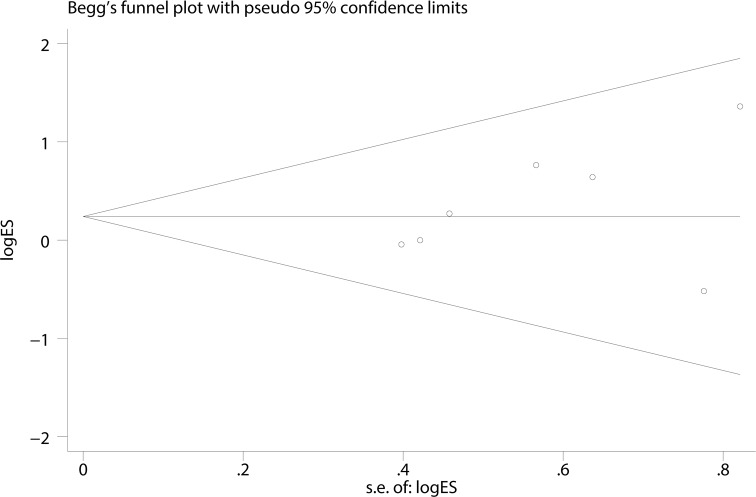
Begg’s funnel plots of the E148Q mutation.

**Fig 4:**
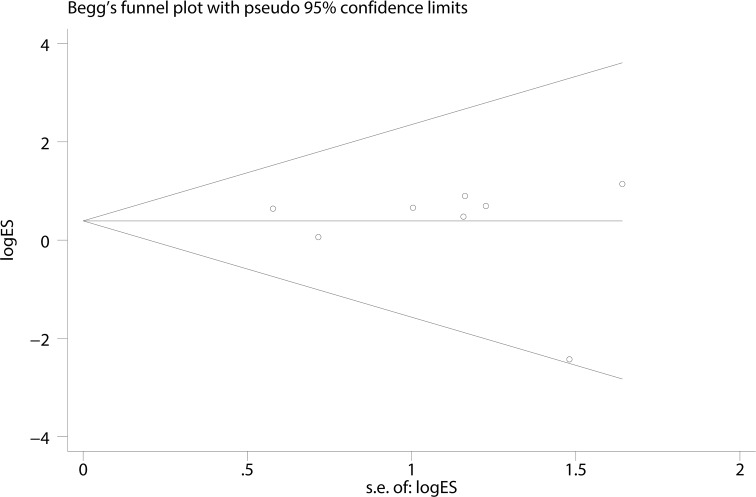
Begg’s funnel plots of the M680I mutation.

**Fig 5:**
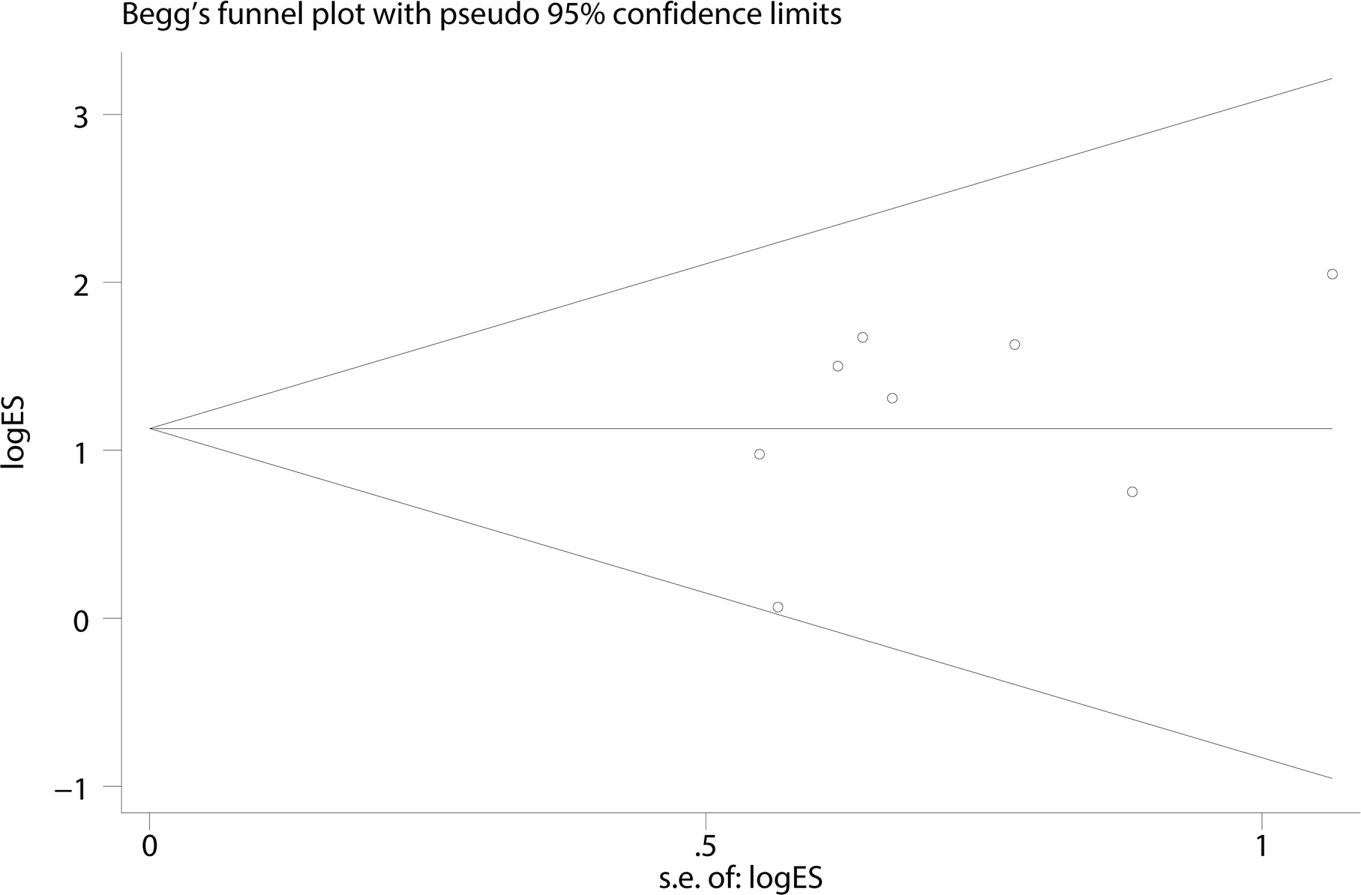
Begg’s funnel plots of the M694V mutation.

**Fig 6:**
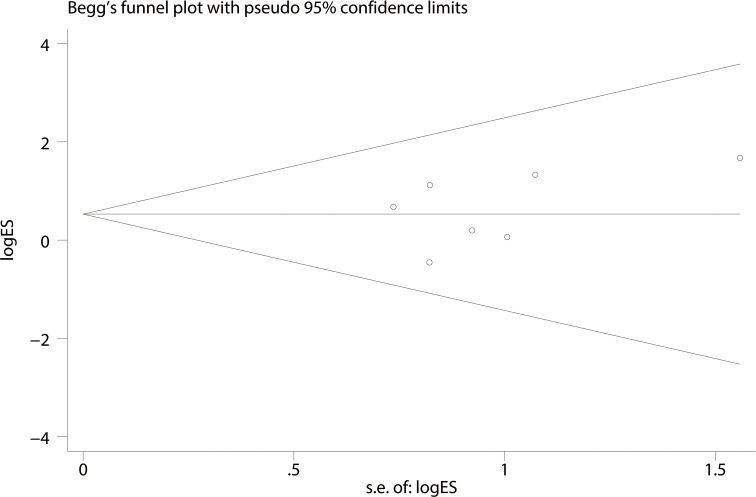
Begg’s funnel plots of the V726A mutation.

**Fig 7:**
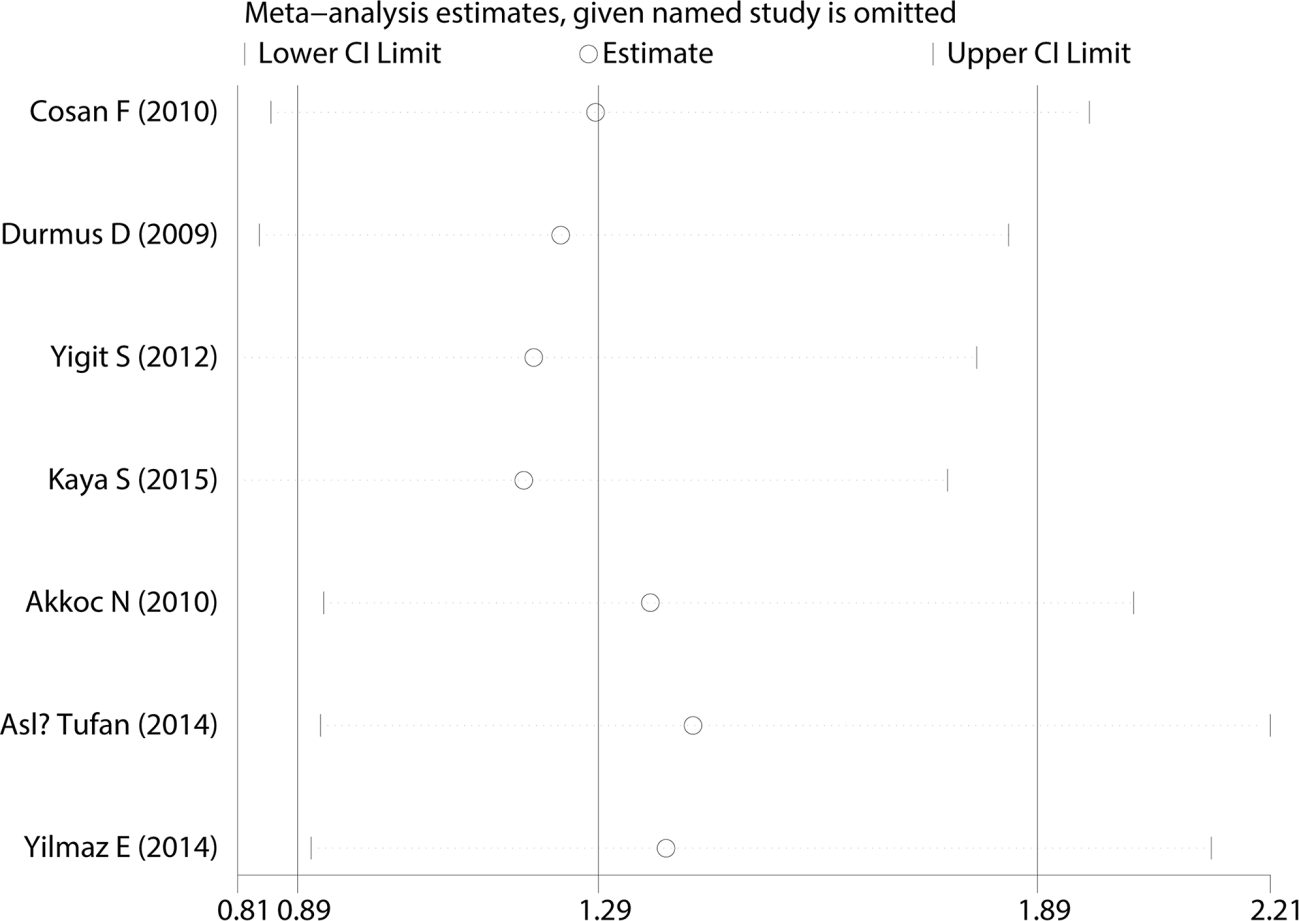
Sensitivity analysis of the E148Q mutation.

**Fig 8:**
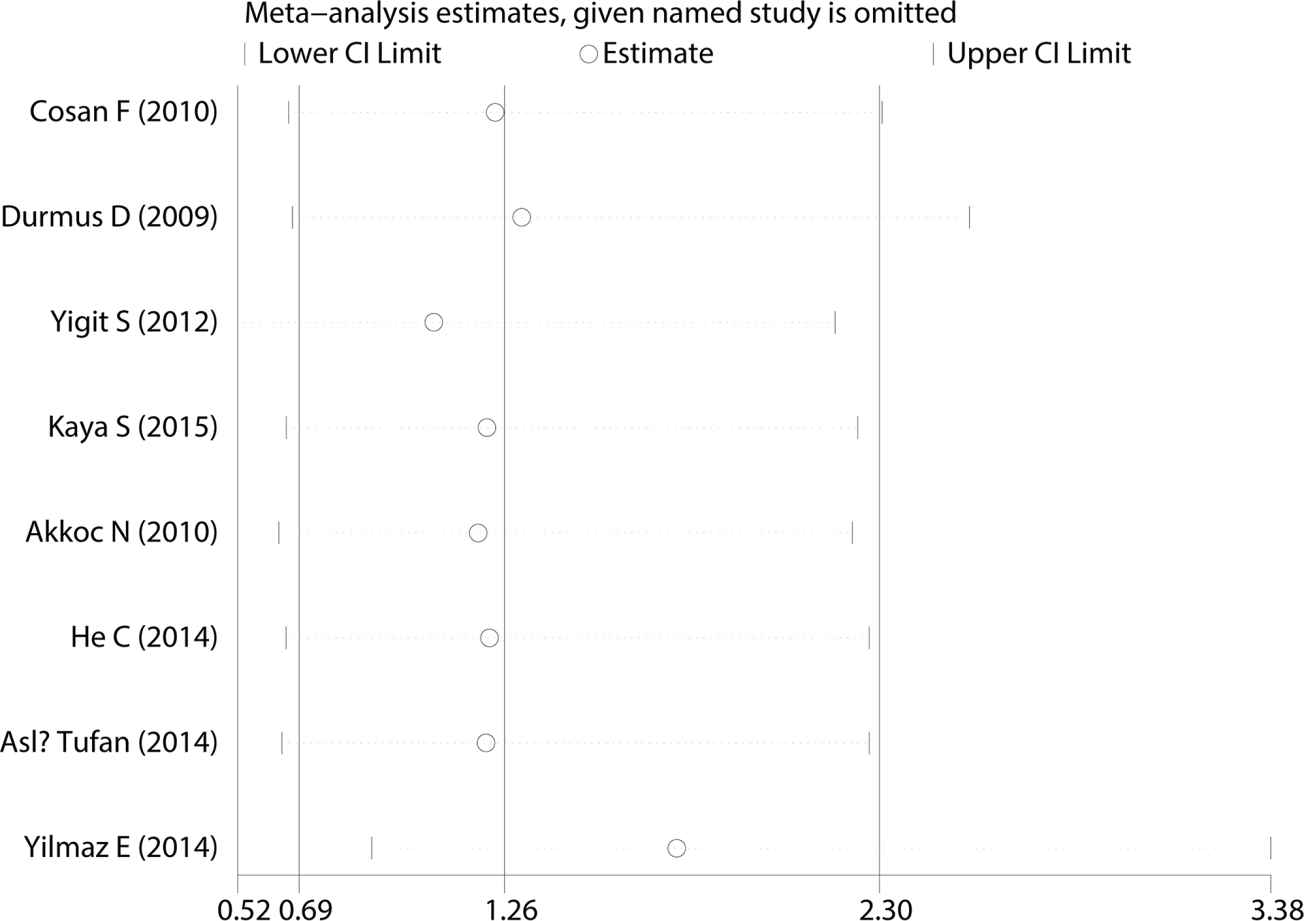
Sensitivity analysis of the M680I mutation.

**Fig 9:**
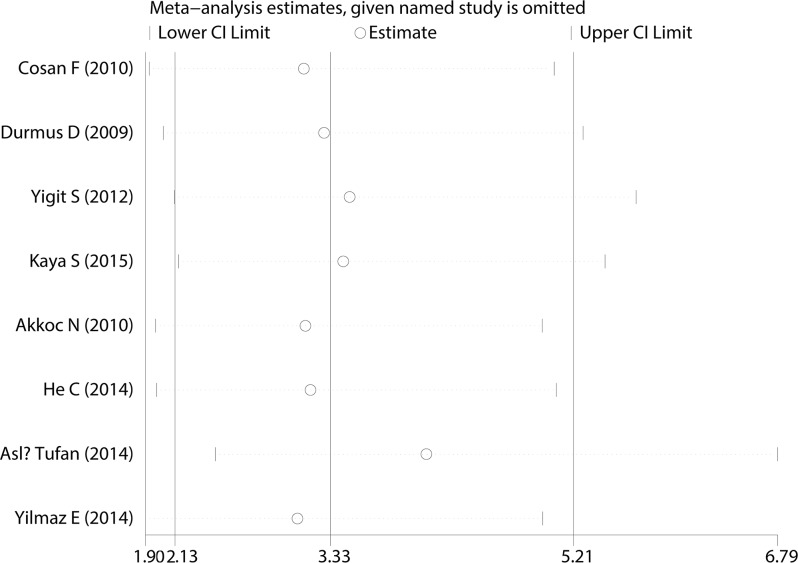
Sensitivity analysis of the M694V mutation.

**Fig 10:**
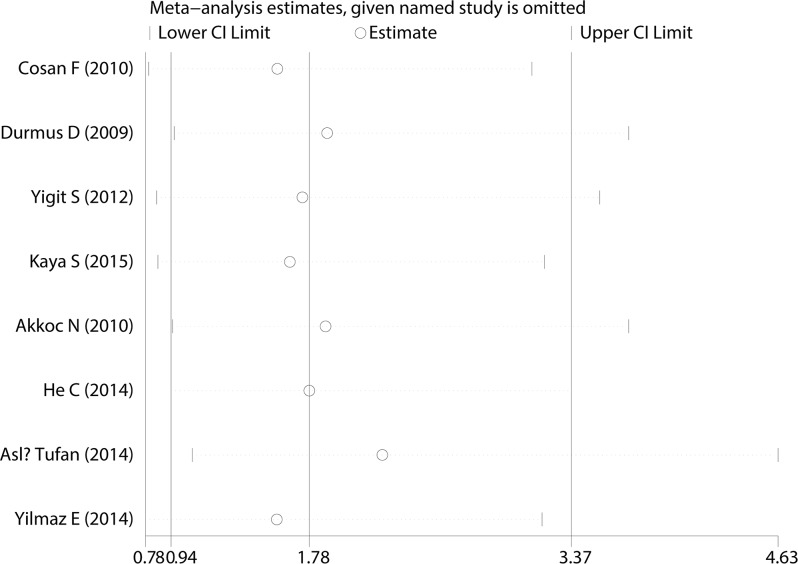
Sensitivity analysis of the V726A mutation.

A Begg’s funnel plot was created to recognize potential publication bias. No significant asymmetry was discovered in the funnel plot (Figs 3–6). Next, an Egger’s test was performed, and the results suggested an absence of publication bias (p = 0.274, 0.597, 0.252, and 0.401). Furthermore, a similar conclusion was reached after the sensitivity analysis, which suggests that the results were stable and reliable (Figs 7–10).

## Discussion

MEFV was originally identified as a candidate gene for familial Mediterranean fever [36, 37]. Its coding protein, *pyrin*, is involved in the assembly of the inflammasome, which is a protein complex that leads to the cleavage and activity of caspase-1 and ultimately results in the activity of the pro-inflammatory cytokine IL-1. Mutations in the MEFV gene are gain of function and thus aggravate inflammatory reactions [38]. Associations have been observed between MEFV and autoimmune diseases, including systemic lupus erythematosus, rheumatoid arthritis and inflammatory bowel disease [39–42].

A whole-genome linkage scan detected a linkage between ankylosing spondylitis and chromosome 16p [27], where MEFV is located. Enthesopathy and sacroiliitis occur in some FMF patients [23–25], and even 7.5% of FMF patients are simultaneously diagnosed with AS [26]. Additionally, several studies have discovered associations of IL-1 polymorphisms with AS [19], which supports the notion that an IL-1-related pathway may play a role in the pathogenesis of AS. Thus, some groups have explored the relationship between MEFV and AS, but the conclusions are uncertain and controversial. Meta-analysis is proposed as an appropriate method to integrate all previous results. Thus, we obtained a relatively comprehensive understanding of the relationship between MEFV and AS.

In this meta-analysis, 8 eligible case-control studies with 869 cases and 879 controls with M680I/M694V and 7 studies with 669 cases and 679 controls with E148Q/V726A were analyzed. Consistent with a previous meta-analysis concerning Behçet's disease [43], a positive connection between AS and M694V was identified. In contrast, no evident relation was present between the other three mutations and AS. Moreover, several studies discovered that MEFV contributed to significantly high bath ankylosing spondylitis disease activity index (BASDAI) values [30, 32], whereas others found no significant differences in the in BASDAIs between the MEFV mutation carriers and non-carriers [31, 35, 44, 45]. MEFV has also been found to be associated with the severity of other autoimmune disorders, such as rheumatoid arthritis [46]. However, due to the high heterogeneity, a meta-analysis regarding AS BASDAI and MEFV is not applicable (data not shown). Additional large scale and reasonably designed research studies are required to confirm their relationship.

To our surprise, the M694V heterozygous mutation was found in two healthy Chinese people. This is inconsistent with the conclusion of the 1000 Genome Project in which the M694V mutation was not discovered in the Chinese population. However, Chinese participants in the 1000 Genome Project were restricted to Xishuangbanna, Beijing, and southern China, while the study of He C [34] concentrated on eastern China. China is a populous country with a vast territory and multiple ethnicities, so there is a great diversity of people in terms of demographic characteristics and genetic backgrounds. Therefore, mutations in exon 10 of MEFV may not be as rare as we previously thought, and more related investigations are needed to verify this point. Secondary amyloidosis is the most severe complication of FMF and occurs in AS patients [47–50]. Additionally, subclinical amyloidosis has been detected in 6.9% of ankylosing spondylitis patients [51], and those who died of secondary amyloidosis account for 12.5% of the total deaths [52]. The regression of AS-related systemic amyloidosis has been reported following the administration of colchicine [53], which is an effective medicine for FMF. As colchicine is economic and effective for the prevention, improvement or even reversal of secondary amyloidosis, it may be worth considering applying this drug to AS.

Another meta-analysis was published concerning the relationship between MEFV and AS [54]. However, the associated literature search was not sufficiently comprehensive, and only 4 studies were included. Additionally, the authors of this study compared the overall MEFV variants of the cases with the controls, but each study detected different mutations. However, there are several limitations in this meta-analysis. First, the subjects were mainly concentrated in Europe and Asia, thus the effect of environment was not assessed. Care should be taken when extending the conclusions to other ethnicities. Consequently, further research from other areas is warranted to reach a more comprehensive conclusion. The second concern is the small sample sizes of some of the studies, which may have caused the statistical power to be inadequate. Third, publication bias can still exist due to the tendency to publish positive results. However, in this meta-analysis, no obvious publication bias was found.

In summary, the current meta-analysis established the potential contribution of the MEFV gene mutation, M694V, to the pathogenesis of ankylosing spondylitis. However, more studies with diverse ethnicities are necessary to validate and extend these conclusions.

## Supporting information

S1 Fig Forest plot of the E148Q mutation. (TIF)

S2 Fig Forest plot of the M680I mutation. (TIF)

S3 Fig Forest plot of the V726A mutation. (TIF)

S1 Table The list of excluded articles and the corresponding reasons for exclusion. (XLS)

S1 File PRISMA checklist. (DOCX)

S2 File The search strategy applied in all databases. (DOCX)

S3 File Meta-analysis of the genetic association studies checklist. (DOCX)
