# Supplementary figures and images for: MEFV M694V mutation has a role in susceptibility to ankylosing spondylitis: A meta-analysis
Source: PLoS One. 2017 Aug 11;12(8):e0182967. doi: 10.1371/journal.pone.0182967 (PMC5553723; doi:10.1371/journal.pone.0182967)

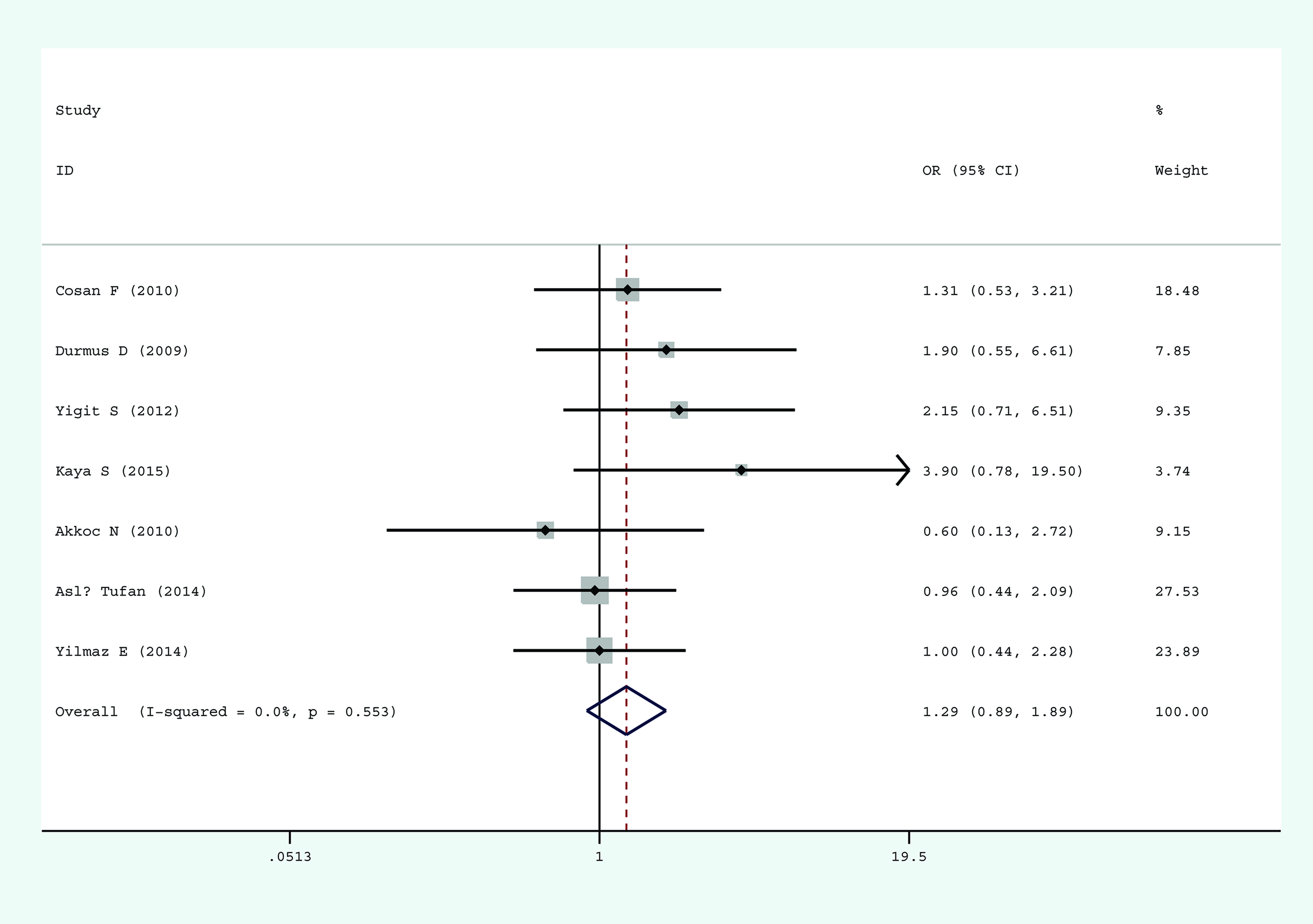

Supplement: S1 Fig — (TIF) [file pone.0182967.s001.tif]

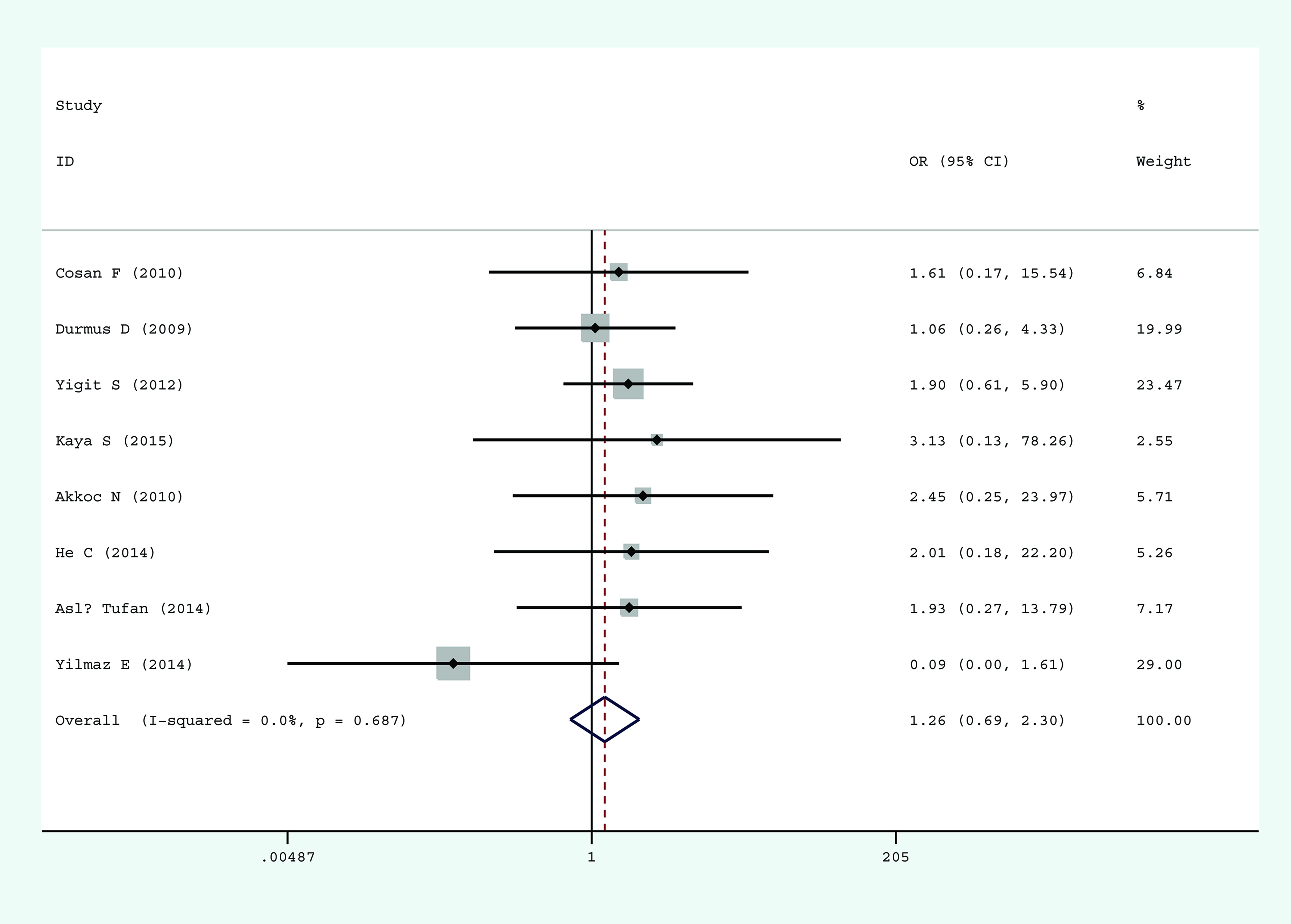

Supplement: S2 Fig — (TIF) [file pone.0182967.s002.tif]

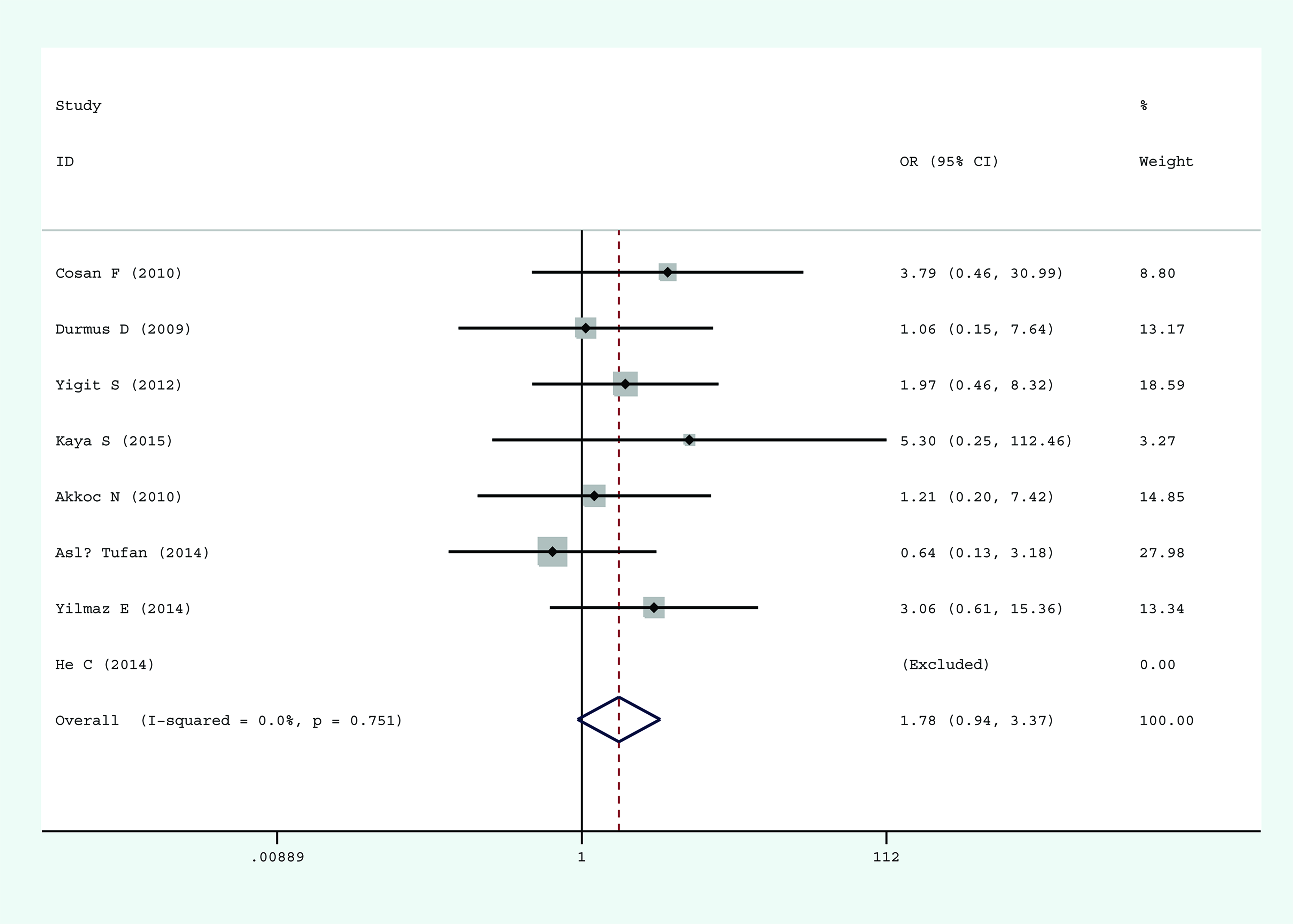

Supplement: S3 Fig — (TIF) [file pone.0182967.s003.tif]
