# Supplementary material for: MEFV M694V mutation has a role in susceptibility to ankylosing spondylitis: A meta-analysis
Source: PLoS One. 2017 Aug 11;12(8):e0182967. doi: 10.1371/journal.pone.0182967 (PMC5553723; doi:10.1371/journal.pone.0182967)
Supplement: S2 File — (DOCX) [file pone.0182967.s006.docx]

The searching strategy is ***“ankylosing spondylitis” and (“Mediterranean fever gene” or MEFV)*.** A comprehensive literature search was performed in PubMed, EMBASE, Web of Science, and Scopus databases up to the date of Dem 31, 2016. And full text search was used in PubMed, EMBASE and Scopus database, while medical subject headings (MeSH) search was applied in Web of Science database. No restrictions were imposed.
